# Investigation of H_2_O_2_ Electrochemical Behavior on Ferricyanide-Confined Electrode Based on Ionic Liquid-Functionalized Silica-Mesostructured Cellular Foam

**DOI:** 10.3390/molecules27249028

**Published:** 2022-12-18

**Authors:** Ling Zhang, Zhenkuan Ma, Yun Fan, Songlin Jiao, Zhan Yu, Xuwei Chen

**Affiliations:** 1 College of Chemistry and Chemical Engineering, Shenyang Normal University of China, Shenyang 110034, China; 2 Research Center for Analytical Sciences, Department of Chemistry, College of Sciences, Northeastern University, Box332, Shenyang 110819, China

**Keywords:** silica mesostructured cellular foam, hydrogen peroxide, ferricyanide, reduction, Prussian blue, ionic liquid

## Abstract

In this work, ionic liquid (IL) of 1-propyl-3-methyl imidazolium chloride-functionalized silica-mesostructured cellular foam (MCF) was prepared. The obtained MCF-IL was used to construct the Fe(CN)_6_^3−^-confined electrode (MCF-IL-Fe(CN)_6_^3−^/PVA) and H_2_O_2_ electrochemical behavior on the electrode was investigated. It was found that H_2_O_2_ was oxidized on the freshly prepared electrode while catalytically electro-reduced on the acid pretreated one. Cyclic voltametric results revealed that the real catalyst for catalytic reduction of H_2_O_2_ was Prussian blue (PB) rather than Fe(CN)_6_^3−^. The electrocatalytic ability of the acid-pretreated MCF-IL-Fe(CN)_6_^3−^/PVA electrode offered a wide linear range for H_2_O_2_ detection. The present study on H_2_O_2_ electrochemical behavior on an MCF-IL-Fe(CN)_6_^3−^/PVA electrode might provide useful information for further developing integrated Fe(CN)_6_^3−^-mediated biosensors as H_2_O_2_ is extensively involved in the classic reaction containing oxidase enzymes.

## 1. Introduction

Silica-based mesoporous materials (including the organic functionalized forms) have been proven promising as electrode matrices in the fields of sensing or biosensing, thanks to their properties of high mechanical and thermal stability, organic functionalized feasibility, good adsorption and penetrability [1]. Rohlfing et al. firstly utilized –NH_2_ or –R–NH_4_^+^ functionalized transparent mesoporous silica films as efficient support to confine electronic mediator such as ferricyanide and revealed the electrochemical characteristics of the resultant electroactive film. They pointed out that the possible charge propagation pathway through the insulating silica matrix was the electron exchange between the adjacent electroactive centers (electron hopping) [2]. Recently, ordered mesoporous materials such as MCM-41, SBA-15, HMS (especially in a functionalized form) as support for immobilization of redox enzyme or protein for electrocatalytic biosensing were also reported [3,4,5]. Compared with the conventional microporous silica with the pore diameter about 1–10 nm, the large pore sized mesoporous silica is highly desired to enhance the adsorption or mass transport properties. Mesostructured cellular foam (MCF) is a large pore-sized silica material with an ultra-large pore size ranging from 15 to 50 nm and possesses continuous 3D pore architecture [6]. Up to now, the reported works about MCF applications in electrochemistry mostly focus on the direct electron transfer behavior of immobilized proteins [7,8,9]. In this respect, further investigations on confining an electronic mediator on functionalized MCF are highly desired, as it could provide useful information for the construction of electronic mediator–biomolecule-integrated sensors in vivo.

Fe(CN)_6_^3−^, as an effective anionic electron mediator, has been widely used in field of electrochemical analysis and biosensing, due to its excellent electron transferability and high degree of reversibility [10,11]. Generally, Fe(CN)_6_^3−^ is dissolved in a detection solution to achieve the signal of electron transfer [12,13]. However, solution-phased Fe(CN)_6_^3−^ may bring the risk of sample contamination, which limits the practical application of Fe(CN)_6_^3−^ as a mediator in electrochemical sensors. The confining of Fe(CN)_6_^3−^ onto electrode matrices with positively charged groups has been proven to be an effective answer to above question, which also shows advantages of shortening analytical time, reducing reagent consumption, simplifying experimental design and constructing integrative electrochemical biosensor [14,15].

As a group of organic salts consisting entirely of ions (anions and cations), ionic liquids (ILs) exist in the form of a liquid at low temperature (<100 °C). The distinctive physiochemical properties such as low vapor pressure [16], high thermal stabilities [17] and broad electrochemical windows [18] have made ILs popular candidates as solvent/additive in various fields [19]. Imidazolium-based ILs are one of the most popular ionic liquids because of their inherent properties and tunable nature. Compared with the commonly used groups (–NH_2_, –R–NH_4_^+^) for Fe(CN)_6_^3−^ confinement [14,20,21], imidazolium-based ILs have recently attracted considerable attention because the strong interaction between the imidazolium moieties and Fe(CN)_6_^3−^ [22]. The characteristics of ion exchange ability of imidazolium-based ILs could be well maintained whether the ILs exist in liquid state or in the form of a functional group. By taking advantage of the ion exchange reaction between the imidazolium moieties of ILs and K_3_Fe(CN)_6_, Fe(CN)_6_^3−^ can be easily entrapped into a sol–gel-modified electrode [23]. Wadhawan et al. prepared water-immiscible IL-modified electrodes and found that the imidazolium-based IL acted as a “sponge” for Fe(CN)_6_^3−^ confinement [24]. Chang et al. reported the fabrication of an Fe(CN)_6_^3−^-confined polymeric imidazolium-based IL-modified electrode and the long-term stability of the as-prepared electrode was especially highlighted [25]. Imidazolium-based polymer IL poly(1-vinyl-3-butylimidazolium chloride) was also used as the matrix for confinement of Fe(CN)_6_^3−^ to construct the integrated ferricyanide-mediated GOD biosensor in vivo [22]. Xiang et al. introduced imidazolium moieties (methylimidazolium, MIM) onto a polyether-grafted multiwalled carbon nanotube for confining Fe(CN)_6_^3−^ and finally constructed an integrative electrochemical biosensor for sensing glucose and O_2_ [26]. Compared with the widely reported imidazolium moieties contained in a poly-IL membrane for confining Fe(CN)_6_^3−^, the utilization of large-pore mesoporous silica as the matrix for functionalization with imidazolium-based ionic liquid would offer the advantage of better absorption and mass transport properties.

H_2_O_2_ exists in a wide range of biological processes, which is generated as a byproduct of an enzyme-catalyzed reaction [22,26,27,28]. As ferricyanide-confined electrodes along with oxidase enzymes have been used in electrochemical biosensing, a fundamental study related to the exact H_2_O_2_ electrochemical behavior on Fe(CN)_6_^3−^-confined electrodes is necessary. Clarifying the electrochemical characteristics of H_2_O_2_ on an Fe(CN)_6_^3−^-confined electrode is helpful to understand sensing mechanisms to achieve better performance, especially regarding in vivo measurement.

In present study, MCF was adopted as the matrix to confine Fe(CN)_6_^3−^, as it is an excellent candidate for the construction of integrated sensors. MCF was firstly prepared via microemulsion templating method, then IL 1-propyl-3-methyl imidazolium chloride was functionalized on the MCF through silanization. Thereafter, an MCF-IL/PVA electrode was prepared using a simple casting method, and Fe(CN)_6_^3−^ was confined on the electrode through ion exchange. Finally, the electrochemical behavior of H_2_O_2_ on both the freshly prepared and acid pre-treated Fe(CN)_6_^3−^-confined electrode (MCF-IL-Fe(CN)_6_^3−^/PVA) was investigated using the cyclic voltammetry technique. Experimental results revealed that H_2_O_2_ could be oxidized on the freshly prepared electrode while being electrocatalytically reduced on the acid pretreated one. In addition, the cyclic voltammogram comparative study revealed that Prussian blue (PB) would generate on the acid-pretreated electrode, which could act as effective catalyst for electroreduction of H_2_O_2_. The electrocatalytic ability of the acid-pretreated electrode to H_2_O_2_ was also investigated and discussed.

## 2. Results and Discussion

### 2.1. Characterizations of Silica Mesostructured Cellular Foam

**Figure 1:**
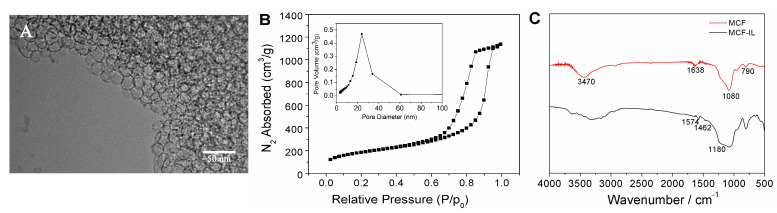
(**A**) TEM micrograph of MCF; (**B**) N_2_ adsorption–desorption isotherms and BJH (inset) and (**C**) FT-IR spectra of MCF and MCF-IL.

Figure 1A shows the TEM image for MCF. It can be seen that MCF consisted of a disordered three-dimensional structure, which is consistent with a previous report [6]. Figure 1B shows the nitrogen adsorption–desorption isotherm and pore size distribution of MCF. The isotherm of MCF was found to be a typical IV curve with significant hysteresis loops, indicating the characteristic of mesoporous structure [6]. The sharp in the nitrogen adsorption–desorption isotherms rose at high relative pressure indicating the existence of large mesopores in the MCF. Determined by the BJH method (inset of Figure 1B), the pore sizes of MCF were calculated ranging from 19 to 30 nm. 

The presence of the imidazolium-based IL group onto the mesoporous framework of post-synthesis of MCF was confirmed by FT-IR spectra. Figure 1C illustrates the FT-IR spectra of MCF and MCF-IL. MCF exhibited characteristic bands at 3470 cm^−1^ belonging to the Si-OH stretching vibration and 1638 cm^−1^ belonging to the O-H stretching vibration. In addition, the unique bands at around 1080, 956, 790 and 565 cm^−1^ were ascribed to the Si-O stretching vibration [29]. For MCF-IL, the above peaks were still maintained even after the subsequent organic modification. The peak of Si-CH_2_-R in the range 1250–1200 cm^−1^ was not resolved due to overlay with the IR absorptions of Si-O-Si in the range 1130–1000 cm^−1^. The bands at 1574 cm^−1^ (imidazolium groups), 1462 cm^−1^ (propyl group) and 619 cm^−1^ (methyl group) attributed to C-H bending vibration were not observed in the spectrum of MCF [30], which further confirmed the functionalization of an imidazolium-based IL group on MCF surface.

**Figure 2:**
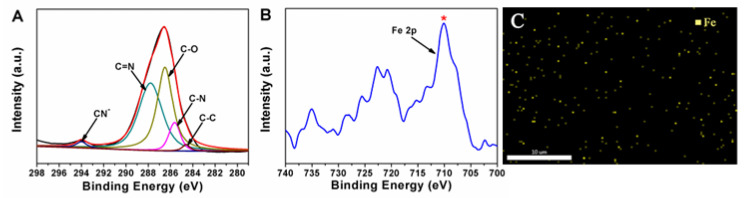
(**A**) The XPS spectra of C 1 s and (**B**) Fe 2p core level of MCF-IL-Fe(CN)_6_^3−^; (**C**) SEM-EDS element-mapping image of Fe in MCF-IL-Fe(CN)_6_^3−^.

The confinement of Fe(CN)_6_^3−^ onto MCF-IL was verified by XPS, energy dispersive spectrometer (EDS) and element mapping analysis. Figure 2A shows the XPS patterns of the C 1s core level of MCF-IL-Fe(CN)_6_^3−^, in which the binding energies of about 284.6, 285.6, 286.5, 287 and 294 eV ascribed to the sp3 C-C, C–N, C–O, C=N bonds and CN^−^ species were clearly observed, indicating the presence of nitrogen-containing functional groups [31]. The binding energy shown in Figure 2B at about 710.9 eV was ascribed to the Fe 2p species, indicating that Fe element was successfully incorporated into the mesostructured cellular foam [32]. Moreover, SEM-coupled element mapping analysis (Figure 2C) revealed that Fe element was uniformly distributed on the functionalized silica surface.

### 2.2. Electrochemical Characterizations of MCF-IL-Fe(CN)_6_^3−^/PVA Electrode

**Figure 3:**
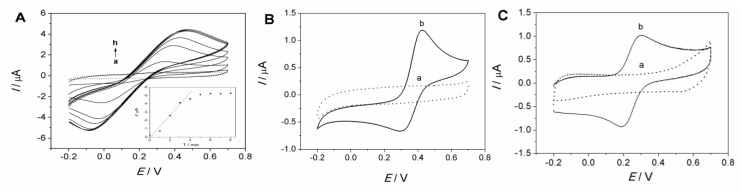
(**A**) Cyclic voltammograms (CVs) of MCF-IL/PVA electrode dipping in 1 × 10^−4^ mol/L Fe(CN)_6_^3−^ solution scanned at every minute. Inset: plots of I_pc_ versus immersion time; (**B**) CVs of (a) MCF-IL/PVA electrode and (b) MCF-IL-Fe(CN)_6_^3-^/PVA electrode obtained in 0.1 M HCl + 0.1 M KCl solution and (**C**) that in 0.1 M PBS + 0.1 M KCl (pH = 7.0). Potential range: −0.2 V~0.7 V; scan rate: 100 mV s^−1^.

The adsorption of Fe(CN)_6_^3−^ onto the MCF-IL/PVA electrode was characterized by cyclic voltammetry technique with electrode dipping in 1 × 10^−4^ mol/L Fe(CN)_6_^3−^ solution. As shown in Figure 3A, the redox peak currents increased evidently with increasing the dipping time, suggesting the adsorption and the continuous confinement of Fe(CN)_6_^3−^ onto the imidazolium-based IL-functionalized MCF. Insets in Figure 3A show the relationship of reduction peak currents of Fe(CN)_6_^3−/4−^ (I_pc_) versus dipping time on the MCF-IL/PVA electrode. As can be seen, the reduction peak current reached the maximum value in 5 min, suggesting that the saturated adsorption state for the MCF-IL/PVA electrode could be achieved in a short period of time. 

After reaching the saturated adsorption of Fe(CN)_6_^3−^ onto the MCF-IL/PVA electrode, the dipped MCF-IL/PVA electrode was taken out from the solution and rinsed with distilled water. Then, the CVs of the resultant electrode and the MCF-IL/PVA electrode without dipping treatment were recorded in pure electrolyte solution in different pH conditions, respectively, as displayed in Figure 3B,C. It can be seen that no obvious redox signals are observed on MCF-IL/PVA electrodes either in HCl solution (curve a in Figure 3B) or in PBS (curve a in Figure 3C), indicating inactivity of the MCF-IL/PVA electrode. On the contrary, the dipped MCF-IL/PVA electrode exhibited a pair of well-defined redox peaks at 289 and 389 mV (curve b in Figure 3B) in 0.1 M HCl solution and a pair of well-defined redox peaks at 164 and 289 mV (curve b in Figure 3C) in PBS (pH = 7.0) at 100 mV/s, indicating the typical redox behavior of Fe(CN)_6_^3−^ confined onto MCF-IL.

### 2.3. Electrochemical Behavior of H_2_O_2_ on the MCF-IL-Fe(CN)_6_^3−^/PVA Electrode

**Figure 4:**
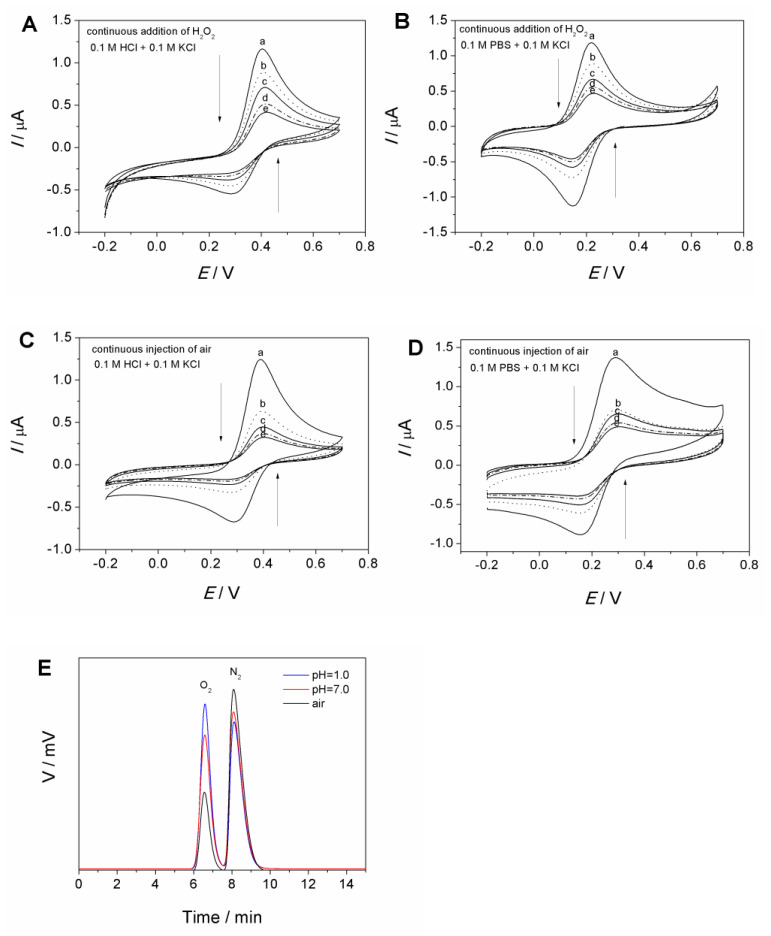
(**A**) CVs of MCF-IL-Fe(CN)_6_^3−^/PVA electrode in 0.1 M HCl + 0.1 M KCl solution and (**B**) that in 0.1 M PBS + 0.1 M KCl solution upon the continuous addition of H_2_O_2_ (a–e); (**C**) CVs of MCF-IL-Fe(CN)_6_^3−^/PVA electrode in 0.1 M HCl + 0.1 M KCl solution and (**D**) that in 0.1 M PBS + 0.1 M KCl solution upon the continuous injection of 10 mL air (a–e). Potential range: −0.2 V~0.7 V; scan rate: 100 mV s^−1^; (**E**) gas chromatogram of air (black line) and gas collected after Fe(CN)_6_^3−^ reaction with H_2_O_2_ in 0.1 M HCl + 0.1 M KCl solution (blue line) and 0.1 M PBS + 0.1 M KCl solution (red line).

Figure 4 shows the electrochemical behaviors of H_2_O_2_ and air on the MCF-IL-Fe(CN)_6_^3−^/PVA electrode in 0.1 M HCl + 0.1 M KCl solution and in 0.1 M PBS + 0.1 M KCl solution (pH = 7.0), respectively. 

As shown in Figure 4A,B, with the addition of H_2_O_2_, the continuous internal contraction in redox currents for Fe(CN)_6_^3−/4−^ was observed whether in HCl solution (Figure 4A) or in PBS (Figure 4B). In addition to H_2_O_2_, it was found that air could also lead to a similar contraction in Fe(CN)_6_^3−/4−^ redox curves (Figure 4C,D).

In order to explore the relationship between H_2_O_2_, O_2_ and Fe(CN)_6_^3−^, the chemical reaction between Fe(CN)_6_^3−^ and H_2_O_2_ was studied. It was found that when H_2_O_2_ was added into the acidic Fe(CN)_6_^3−^ solution, a larger number of fine bubbles appeared compared with the solution without Fe(CN)_6_^3−^. Taking into account of the experimental conditions, we inferred that the generated gas was O_2_, resulting from the oxidation of H_2_O_2_ as the following reaction.

(1) $$H_{2}O_{2}\overset{{\mathrm{Fe}(\mathrm{CN})}_{6}^{3-}}{\to }H_{2}O+O_{2}↑$$

To verify this speculation, gas chromatography was adopted to ascertain the gaseous production. As shown in Figure 4E, the initial gas in the reaction vessel was air containing O_2_ and N_2_. After H_2_O_2_ was added into the to the acidic and neutral Fe(CN)_6_^3−^ solution, the gas chromatography signal for O_2_ both increased obviously, demonstrating the production of O_2_ following the above reaction.

The above phenomena demonstrated that Fe(CN)_6_^3−^ confined on the MCF-IL-Fe(CN)_6_^3−^/PVA electrode could react with H_2_O_2_ spontaneously and quickly, leading to O_2_ generation. Therefore, the electrochemical response of H_2_O_2_ on the MCF-IL-Fe(CN)_6_^3−^/PVA electrode’s behavior was consistent with that of O_2_ on the MCF-IL-Fe(CN)_6_^3−^/PVA electrode. However, the relevant mechanism for the contraction in Fe(CN)_6_^3−/4−^ CVs in the presence of O_2_ still remains unclear, which is worthy of further deep study.

### 2.4. Electrochemical Response of H_2_O_2_ on the Acid-Pretreated MCF-IL-Fe(CN)_6_^3−^/PVA Electrode

**Figure 5:**
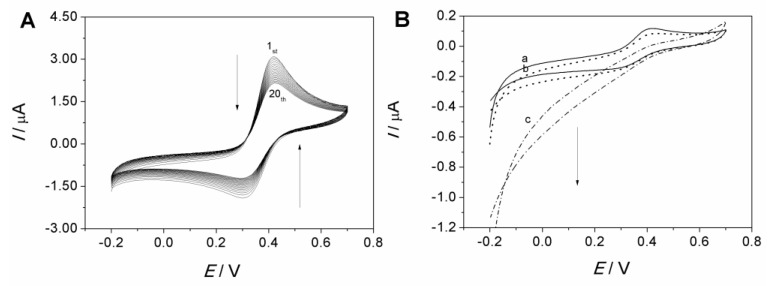
(**A**) Twenty consecutive CVs obtained on MCF-IL-Fe(CN)_6_^3−^/PVA electrode in 0.1 M HCl + 0.1 M KCl solution, scan rate: 100 mV s^−1^; (**B**) CVs obtained on acid-pretreated MCF-IL-Fe(CN)_6_^3−^/PVA electrode upon the continuous addition of H_2_O_2_: (a–c); scan rate: 10 mV s^−1^.

Figure 5A shows the 20 consecutive cyclic voltammograms obtained on the freshly prepared MCF-IL-Fe(CN)_6_^3−^/PVA electrode in 0.1 M HCl + 0.1 M KCl solution. With cycling, the redox currents of Fe(CN)_6_^3−/4−^ exhibited continuous decrease. As the IL 1-propyl-3-methyl imidazolium chloride was covalently grafted on MCF via silylation reaction, it was stable without chance of leakage [33]. So, the decrease in redox current on the MCF-IL-Fe(CN)_6_^3−^/PVA electrode was deduced from the change in Fe(CN)_6_^3−^ amount.

In reported studies, the decreasing currents on the Fe(CN)_6_^3−^-confined electrode was also observed, and the electrode was treated with acid solution to obtain a stable current signal [16]. However, Xia et al. and Yang et al. reported that Fe(CN)_6_^3−^ was unstable in acidic conditions [34,35]. Thus, the acid pretreatment experiment for the MCF-IL-Fe(CN)_6_^3−^/PVA electrode was performed to see if the characteristics of the electrode were altered. In this work, the acid-pretreated MCF-IL-Fe(CN)_6_^3−^/PVA electrode was prepared by potentially scanning the MCF-IL-Fe(CN)_6_^3−^/PVA electrode in potential range of −0.2 V~0.7 V in 0.1 M HCl + 0.1 M KCl solution until its currents reached the constant value and was then washed with 0.1 M HCl solution.

The cyclic voltammograms of the acid-pretreated MCF-IL-Fe(CN)_6_^3−^/PVA electrode in absence and presence of H_2_O_2_ in 0.1 M HCl + 0.1 M KCl solution are illustrated in Figure 5B. It can be seen that the treated electrode still presented a pair of reductive–oxidative peaks in absence of H_2_O_2_ (curve a). With addition of H_2_O_2_, the reductive peak current increased and the oxidative peak current decreased (curves b and c), indicating the occurrence of electroreduction of H_2_O_2_ on the electrode [20]. By comparing the differences in cyclic voltametric responses of H_2_O_2_ on the freshly prepared MCF-IL-Fe(CN)_6_^3−^/PVA electrode and the acid-pretreated one, it can be seen that the acid treatment greatly affected the characteristics of the electrode.

**Figure 6:**
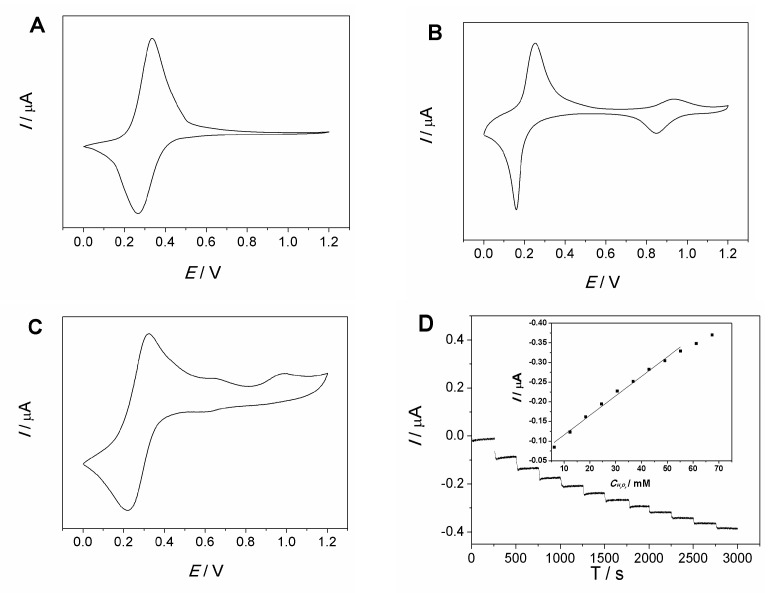
(**A**) CVs obtained on a freshly prepared MCF-IL-Fe(CN)_6_^3−^/PVA electrode, (**B**) PB electrode and (**C**) acid-pretreated MCF-IL-Fe(CN)_6_^3−^/PVA electrode in 0.1 M HCl + 0.1 M KCl solution. Potential range: 0.0 V~1.2 V; scan rate: 10 mV s^−1^; (**D**) chronoamperometric curve for the acid-pretreated MCF-IL- Fe(CN)_6_^3−^/PVA electrode for successive addition of 6.12 mM H_2_O_2_ every 250 s in 0.1 M HCl + 0.1 M KCl solution at 0.2 V. Inset: calibration plot of MCF-IL-Fe(CN)_6_^3−^/PVA electrode for H_2_O_2_ detection.

It has been demonstrated that Fe(CN)_6_^3−^ could decompose in acidic conditions and generate PB on the electrode surface [34,35]. In order to confirm the generation of PB on the acid-pretreated MCF-IL-Fe(CN)_6_^3−^/PVA electrode, a cyclic voltammogram comparative study was performed. Figure 6 shows the CVs on a freshly prepared MCF-IL-Fe(CN)_6_^3−^/PVA electrode, a PB electrode and an acid-pretreated MCF-IL-Fe(CN)_6_^3−^/PVA electrode in 0.1 M HCl + 0.1 M KCl solution scanned within a wide potential range, respectively. 

Figure 6A shows the CV on a freshly prepared MCF-IL-Fe(CN)_6_^3−^/PVA electrode, where a pair of well-defined reductive–oxidative peaks at about 0.33 V were observed, indicating the typical redox behavior of Fe(CN)_6_^3−/4−^. Figure 6B shows the CV on a PB electrode, which presented two couples of redox peaks with formal potential around 0.20 V and 0.95 V, corresponding to the typical cyclic voltammograms of PB [36]. The couple of redox peaks with formal potential around 0.20 V indicated the transformation between Prussian blue and Prussian white (PB/PW), while the couple of redox peaks around 0.95 V presents the transformation between Prussian blue and Berlin green (PB/PG). Figure 6C displays the CV on the acid-pretreated MCF-IL-Fe(CN)_6_^3−^/PVA electrode. A pair of redox peaks with a formal potential value of 0.27 V and an anodic peak at 0.95 V was observed.

Compared with the CVs of Fe(CN)_6_^3−/4−^ on the freshly prepared electrode, an additional anodic peak at 0.95 V on the acid-pretreated one was observed, suggesting some other substrate was generated, and the peak was close to the anodic peak of PB electrode at approximately 0.95 V, indicating that a small amount of PB was generated. Here, only an anodic peak for PB/PG was observed on the acid-treated electrode, which may be ascribed to the fact that the dissociation rate of Fe(CN)_6_^3−^ was slow, and only a part of Fe(CN)_6_^3−^ dissociated and subsequently converted into PB. According to the above statement, the pair of redox peaks for PB located at 0.20 V on the acid-pretreated electrode also existed. This was verified by the presence of the redox peaks loaded at 0.27 V on the treated electrode, which was a negative shift compared to the redox peaks for Fe(CN)_6_^3−/4−^ (0.33 V) on the freshly prepared MCF-IL-Fe(CN)_6_^3−^/PVA electrode. The negative shift was attributed to the overlap of the redox peaks for Fe(CN)_6_^3−/4−^ (0.33 V) and PB/PW (0.20 V). Considering the slow dissociation rate of Fe(CN)_6_^3−^, it was reasonable to deem that PB and Fe(CN)_6_^3−^ coexisted on the acid-pretreated electrode. When H_2_O_2_ was added in the solution, the acid-pretreated electrode showed some characteristics of a PB-modified electrode to reduce H_2_O_2_ at low scan rate, as shown in Figure 5B. This cyclic voltammogram comparative study further proved that on the acid pre-treated Fe(CN)_6_^3−^-confined electrode, the real catalyst for electroreduction of H_2_O_2_ was PB rather than Fe(CN)_6_^3−^.

The electrocatalytic ability of the acid-pretreated MCF-IL-Fe(CN)_6_^3−^/PVA electrode to H_2_O_2_ was also investigated as displayed in Figure 6D, where the chronoamperometry response curve of the electrode upon successive additions of H_2_O_2_ and the inset of the calibration plot for H_2_O_2_ detection were included. It demonstrated that the linear response range was from 6.13 mM to 55.08 mM, with a sensitivity of 74 μA cm^−2^ mM^−1^.

Table 1 summarizes the sensing performance of the as-prepared electrode with reported electrodes for H_2_O_2_ sensors. Compared with other electrodes, the acid-pretreated MCF-IL-Fe(CN)_6_^3−^/PVA electrode exhibited wider linear detection range for H_2_O_2_ detection owing to the favorable mass transport condition, which makes it more attractive in practical assay as the H_2_O_2_ level in real samples varies greatly.

## 3. Materials and Methods

### 3.1. Materials

Potassium ferricyanide (K_3_Fe(CN)_6_), hydrochloric acid (HCl), polyvinyl alcohol (PVA), H_2_O_2_ and ethanol were purchased from Sinopharm Chemical Reagent Co., Ltd. (Shenyang, China) Tetraethoxysilane (TEOS), pluronic 123 (P123) and 1,3,5-trimethylbenzene (TMB) were obtained from Sigma (Los Angeles, CA, USA). The grafting reagent 3-chloropropyl triethoxysilane was purchased from Merck (Darmstadt, Germany) and N-methylimidazole was obtained from Aladdin (Shanghai, China). All reagents were of the highest grade available and used without further purification. All aqueous solutions were prepared with ultra-pure water obtained from the Milli-Q (18.3 MΩ cm^−1^) water system.

### 3.2. Methods

IR spectra of pure MCF and MCF-IL were recorded separately on a Nicolet 380 spectrophotometer (KBr pellets in the range of 400–4000 cm^−1^). The specific surface area and the pore volume were measured using the ASAP-2010C adsorption meter. Morphologies of the samples were investigated by using a scanning electron microscope (SEM, Ultra Plus, Carl Zeiss, Jena, Germany) and transmission electron microscopy (TEM, JEM-2100, JEOL, Tokyo, Japan) operating at 120 kV. Energy dispersive spectrometry (EDS) was obtained on SEM instrument with an acceleration voltage of 20 kV. X-ray photoelectron spectroscopy (XPS) analysis was performed on an XPS spectrometer (ESCALAB 250Xi, Thermo Fisher Scientific, USA) with Al Kα radiation (λ = 8.34 A) as the excitation source. The generation of oxygen was measured by a gas chromatography 689 A using a thermal conductivity detector and argon as the carrier gas. Electrochemical experiments were carried out on an Autolab electrochemical station (Autolab, Utrecht, The Netherlands). All of the electrochemical experiments were performed with a conventional three-electrode system. Ag/AgCl (saturated KCl) was used as the reference electrode. The Pt wire acted as the counter electrode, and the mesoporous silica-modified electrodes acted as the working electrodes.

### 3.3. Synthesis and Functionalization of MCFs

MCF was synthesized according to the previously described procedures [6]. The pore volume of MCF was 1.8 cm^3^/g and the BET surface area was 416.6 m^2^ g^−1^ with pore diameter ranging from 19 to 30 nm. MCF-IL was prepared according to reported procedures [44,45]. For the post-synthesis of ionic liquid-functionalized MCF, firstly, 2.0 g of calcined MCF was refluxed with 12 mL of (3-chloropropyl) trimethoxysilane in 500 mL of anhydrous toluene protected by N_2_ for 24 h. The superfluous (3-chloropropyl) trimethoxysilane was removed by Soxhlet extraction using a mixed solvent of 1/1 diethyl ether and dichloromethane. Secondly, the resulting chlorinated MCF precursor with N-methylimidazole was vigorously stirred in anhydrous toluene at 80 °C for 24 h. After filtering, the resulting material was washed by Soxhlet extraction with acetone as the solvent and MCF-IL was obtained by drying under vacuum at 70 °C overnight. MCF-IL-Fe(CN)_6_^3−^ composite for samples of SEM and XPS was prepared by dispersing 10 mg MCF into 1 mL 1 × 10^−4^ M K_3_Fe(CN)_6_ solution under vortexing and stood for 15 min. Then, the resulting composite was centrifuged and washed with distilled water for three times. After vacuum drying, a pale yellow solid powder of MCF-IL-Fe(CN)_6_^3−^ was obtained.

### 3.4. Preparation of the Electrodes

Before modification, a glass carbon (GC) electrode (Φ = 3 mm) was polished sequentially with 1.0, 0.3 and 0.05 μm Al_2_O_3_, rinsed thoroughly with deionized water after each polishing step, sonicated in deionized water and ethanol successively, and then allowed to dry at room temperature. The MCF-IL/PVA electrode was prepared using a simple casting method. The casting solvent was obtained by mixing 300 μL of the suspension of MCF-IL (1.5 mg mL^−1^) and 15 μL of the PVA solvent (2%). Next, 5 μL of this casting solvent was dropped on the surface of the GC electrode to obtain the MCF-IL/PVA modified electrode, which was then left to dry overnight at room temperature. 

The freshly-prepared MCF-IL-Fe(CN)_6_^3−^/PVA electrode was prepared by immersing the MCF-IL/PVA-modified electrode in a 1 × 10^−4^ M K_3_Fe(CN)_6_ solution until saturation.

The acid-pretreated MCF-IL-Fe(CN)_6_^3−^/PVA electrode was prepared by potentially scanning the MCF-IL-Fe(CN)_6_^3−^/PVA electrode in 0.1 M HCl + 0.1 M KCl solution until its currents reached a constant value and then was washed using 0.1 M HCl solution three times.

All electrochemical measurements were carried out in a 0.1 M HCl + 0.1 M KCl solution or 0.1 M PBS containing 0.1 M KCl (pH = 7.0).

## 4. Conclusions

In the present study, we prepared IL of 1-propyl-3-methyl imidazolium chloride-functionalized MCF silica material. The as-prepared MCF-IL was used to fabricate MCF-IL/PVA electrode via simple casting method; the confinement of Fe(CN)_6_^3−^ on the electrode was achieved through the strong interaction between imidazolium moieties and Fe(CN)_6_^3−^. The electrochemical behavior of H_2_O_2_ on the freshly prepared Fe(CN)_6_^3−^-confined electrode (MCF-IL-Fe(CN)_6_^3−^/PVA) was quite different from that on the acid-pretreated one. H_2_O_2_ was oxidized on the freshly prepared electrode, while catalytically electro-reduced on the acid-pretreated one owing to the generation of Prussian blue.

Since ferricyanide-confined electrodes along with oxidase enzymes have been used in integrated biosensor research, especially for in vivo measurement, the present study about H_2_O_2_ electrochemical behavior on the MCF-IL-Fe(CN)_6_^3−^/PVA electrode might provide useful information to clarify the H_2_O_2_ effect on the performance of the biosensor, as H_2_O_2_ is extensively involved in the classic reaction containing oxidase enzymes.

## Figures and Tables

**Table 1:** Comparison of the sensing performance to H_2_O_2_ for the PB modified electrodes in the literature.

Electrode	Linear Range (mM)	Ref.
Acid-pretreated MCF-IL-Fe(CN)_6_^3−^/PVA	6.125~55.08	this work
Acid-pretreated MCM-41 ^1^-NH_2_-Fe(CN)_6_^3−^/CPE ^2^	1~30	[20]
PB/[Bmim] [Cl] ^3^/GC	5.0~30	[37]
Prussian blue based nanoelectrode arrays	0.01~10	[38]
AuNPs-PB-GO ^4^/GCE	0.0038~5.4	[39]
PPY/MWCNTs/PB ^5^	0.005~0.503 1.403~5.103	[40]
PB/Au CDtrode ^6^	0.001~1.2	[34]
PB-fCNT/TiO_2_.ZrO_2_^7^	0.1~1.0	[41]
ITO/LbL ^8^-CMC ^9^:PANI ^10^:PB	0.002~0.165	[42]
GE ^11^/PBNPs/Nafion	0.0021~0.14	[43]

^1^ MCM-41: MCM-41 mesoporous silica; ^2^ CPE: carbon paste electrode; ^3^ [Bmim] [Cl]: 1-butyl-3-methylimidazolium chloride; ^4^ GO: graphene oxide; ^5^ PPY/MWCNTs/PB: multiwalled carbon nanotubes/Prussian blue–functionalized polypyrrole nano-wire array; ^6^ Au CDtrode: gold CDtrode; ^7^ TiO2.ZrO2-fCNTs: titanium dioxide and zirconia doped functionalized carbon nanotubes; ^8^ LbL: layer-by-layer; ^9^ CMC: carboxymethyl cellulose; ^10^ PANI: polyaniline; ^11^ GE: graphite electrode.

## Data Availability

The data presented in this study are available upon request from the corresponding author.

## Sample Availability

Samples of the compounds are available from the authors.
